# Interleukin-10 production by tumor infiltrating macrophages plays a role in Human Papillomavirus 16 tumor growth

**DOI:** 10.1186/1471-2172-11-27

**Published:** 2010-06-07

**Authors:** Aline Bolpetti, João S Silva, Luisa L Villa, Ana Paula Lepique

**Affiliations:** 1 Fundação Antônio Prudente; Rua Prof. Antonio Prudente, 409, São Paulo, SP, 01509-010, Brazil; 2 Department of Biochemistry and Immunology, School of Medicine of Ribeirão Preto, University of São Paulo; Avenida Bandeirantes, 3900, Campus da USP, Fazenda Monte Alegre, Ribeirão Preto, SP, 14049-900, Brazil; 3 Ludwig Institute for Cancer Research; Rua João Julião, 245, 1o andar, São Paulo, SP, 01323-903, Brazil

## Abstract

**Background:**

Human Papillomavirus, HPV, is the main etiological factor for cervical cancer. Different studies show that in women infected with HPV there is a positive correlation between lesion grade and number of infiltrating macrophages, as well as with IL-10 higher expression. Using a HPV16 associated tumor model in mice, TC-1, our laboratory has demonstrated that tumor infiltrating macrophages are M2-like, induce T cell regulatory phenotype and play an important role in tumor growth. M2 macrophages secrete several cytokines, among them IL-10, which has been shown to play a role in T cell suppression by tumor macrophages in other tumor models. In this work, we sought to establish if IL-10 is part of the mechanism by which HPV tumor associated macrophages induce T cell regulatory phenotype, inhibiting anti-tumor activity and facilitating tumor growth.

**Results:**

TC-1 tumor cells do not express or respond to IL-10, but recruit leukocytes which, within the tumor environment, produce this cytokine. Using IL-10 deficient mice or blocking IL-10 signaling with neutralizing antibodies, we observed a significant reduction in tumor growth, an increase in tumor infiltration by HPV16 E7 specific CD8 lymphocytes, including a population positive for Granzyme B and Perforin expression, and a decrease in the percentage of HPV specific regulatory T cells in the lymph nodes.

**Conclusions:**

Our data shows that in the HPV16 TC-1 tumor mouse model, IL-10 produced by tumor macrophages induce regulatory phenotype on T cells, an immune escape mechanism that facilitates tumor growth. Our results point to a possible mechanism behind the epidemiologic data that correlates higher IL-10 expression with risk of cervical cancer development in HPV infected women.

## Background

High risk human papillomavirus, HR-HPV, is the main etiologic factor for cervical cancer and for a percentage of other anogenital and oropharyngeal tumors [1].

Immune responses against HPV antigens eliminate most of the infections and precursor lesions in women. Moreover, only a percentage of the infected women show persistent infection that leads to malignant disease [2,3]. Persistent infection is inevitably linked to immune evasion mechanisms. HPV display several mechanisms for evading the host's immune system, for example: maintenance of low viral protein levels in the cell, expression of capsid proteins only in external layers of the epithelium and therefore out of reach of antigen presenting cells [4], inhibition of interferon responsive element [5,6], and production of regulatory cytokines like TGFβ[7,8]. Moreover, tumor cells also display evasion mechanisms which, in the case of HPV associated tumor cells, are not well established. One cytokine which expression has been associated with HPV related disease is IL-10 [9-11]. IL-10 is a pleiotropic cytokine produced by myeloid cells and lymphocytes that displays both immunoregulatory and immunostimulatory effects [12]. IL10 inhibits the production of other cytokines such as Interleukin-2 (IL-2), Interferon γ (IFNγ), Interleukin-12 (IL-12), Tumor Necrosis Factor α (TNFα) and it is also associated to Major Histocompatibility Complex-I (MHC-I) downregulation [13,14] resulting in reduction of Th1 response. Different studies have reported increased IL-10 serum levels in patients with melanoma [15] and other solid tumors [16], as well as expression of IL-10 by tumor cells [17-19]. In cervical cancer patients, IL-10 is secreted by regulatory CD4 lymphocytes stimulated with HPV antigens [20]. Higher expression of IL-10 in cervical tissue correlates with higher grade lesions [21-23]. Furthermore, polymorphisms in IL-10 gene promoter have been associated with susceptibility to precursor lesions associated to HPV infection as well with infection clearance [24,25].

Tumor associated macrophages and myeloid derived suppressor cells express IL-10, among other cytokines, as part of the mechanism of suppression of T cell anti-tumor responses [26-28]. Interestingly, increased numbers of macrophages per lesion area have been associated with higher grade cervical disease [29-31].

Our laboratory has been investigating the role of tumor associated myeloid cells in the HPV16 mouse tumor model, TC-1. We have previously shown that TC-1 tumors are infiltrated mainly by CD45^+^CD11b^+^F4/80^+^Arginase1^+ ^macrophages, as well as some CD45^+^CD11b^+^Gr1^+ ^cells [32]. In the present study we tested the hypothesis that IL-10 is part of the mechanism by which tumor infiltrating macrophages inhibit anti-tumor T cell activity [32]. We injected TC-1 cells in IL-10 deficient mice or mice treated with anti-IL10 and anti-IL-10R neutralizing antibodies. In these mice, tumors exhibited delayed growth kinetics and higher infiltration by CD4 and CD8 lymphocytes, including E7 HPV16 specific CTL Perforin^+ ^cells. Furthermore, we observed that IL-10 is necessary for the expansion of B cells in the lymph nodes of tumor bearing mice. Altogether, our results point to IL-10 as one of the elements involved in tumor immune evasion via T cell regulatory phenotype induction.

## Methods

### Mice and cell lines

C57Bl/6 and C57Bl/6 IL-10^*tmlCgn *^(B6.129P2-Il10^*tm1Cgn*^/J #002251) mice [33] were maintained in standard mouse facility at the Faculdade de Medicina, Universidade de São Paulo (Ribeirão Preto, SP, Brazil) with irradiated food and autoclaved water, with cycles of light and dark of 12 hours.

Alternatively, C57Bl/6 mice used for the neutralizing antibodies assays were maintained at the Ludwig Institute for Cancer Research mouse facility in *spf *(specific pathogen free) conditions. All mouse protocols were approved by the Ethics Committee for Animal Experimentation of Fundação Antônio Prudente, São Paulo, Brazil, protocol 05/05.

TC-1 cell line was kindly donated by Dr. TC Wu (John Hopkins, Baltimore, MD). This cell line is derived of lung epithelium transformed with HPV16 E6 and E7 oncogenes and Ha-ras [34]. These cells were cultivated in RPMI supplemented with 10% bovine fetal serum and 400 μg/ml Neomicin (Invitrogen, Carlsbad, CA) in incubators with atmosphere of 5% CO2.

### Tumor formation

TC-1 cells were suspended in Phosphate Buffered Saline, PBS^++ ^(1 mM CaCl_2_, 0.5 mM MgCl_2_) at a concentration of 10^5^cells/100 μl of suspension. Each mouse was injected with 10^5^ TC-1 cells subcutaneously in the right dorsal flank. Tumor formation and size was measured with a caliper until a maximum diameter of 1.5 cm. Mice were observed and measured with intervals of 2 or 3 days from the day when they were injected. Tumor volume was calculated using the formula V = D*d^2^/2, where V is the tumor volume, D is the largest tumor measured diameter and d is the smallest measured tumor diameter.

### Neutralizing antibody treatment

Mice received intraperitoneal doses of 10 mg of anti-IL-10 antibody (mAb417 R&D Systems, Minneapolis, MN) alternated with intraperitoneal doses of 10 μg of anti-IL-10R antibody (AF-474-NA, R&D Systems, Minneapolis, MN) spaced by 4 days. The first dose was given in the same day as the TC-1 cells injection and it was a double dose of anti-IL-10 and anti-IL-10R. Alternatively, mice were treated with 500 μg of protein G purified antiIL10R clone 1B13a. As controls, mice were treated with equal amounts of rat IgG, catalog number 6-001-A (R&D Systems, Minneapolis, MN) or catalog number I4131 (Sigma-Aldrich, St Louis, MO).

### Cell preparations

All cell preparations were performed in 1× Hanks' buffered saline solution supplemented with 5% fetal bovine serum and 0.5 U/ml DNase.

For tumor preparations, these were harvested after mouse euthanasia, a fraction was frozen in Tissue-Tek OCT (Sakura Finetek Europe, NL) for immunofluorescence studies, the rest of the tumor was minced and digested in 100 μg/ml Collagenase under agitation at 37°C for 1 to 2 hours. The cell suspension was filtered through a 70 μm mesh and washed twice before use. Spleen and lymph node suspensions were made by mechanical dissociation of the tissue and filtration through nylon mesh. Splenocytes were submitted to hypotonic cell lyses to eliminate red cells. Peritoneal macrophages were harvested after mouse euthanasia; by injecting 5 ml ice cold PBS into the peritoneal cavity and aspiration of the solution containing the resident cells. After centrifugation, cells were ressuspended in Hanks' buffer, counted and used for macrophage sorting.

### FACS analysis

Single cell suspensions were incubated with the antibodies indicated in each figure. Tumor and spleen suspensions were blocked with Fc ligand (CD16/CD32, clone 2.4G2) (BD Biosciences, San Diego, CA) before incubation with the specific antibodies. The antibodies used in this work were anti-CD4 (cloneGK1.5), anti-CD8 (clone 53-6.7), antiGr1 (clone RB6-8C5), anti-CD11b (clone M1/70), anti-CD19 (clone 1D3), anti-CD45 (clone 30-F11), anti-IL-10 (clone JES5-16E3) purchased from BD Biosciences (San Diego, CA), PE-Cy5.5 conjugated F4/80 (clone BM8) and APC conjugated anti-Perforin were purchased from eBiosciences (San Diego, CA). MHC-I tetramers containing the HPV16 E7_49-57 _epitope or an irrelevant peptide were a kind donation from Dr. Immanuel Luescher and Dr. Philippe Guillaume (Ludwig Institute for Cancer Research, Lausanne, Switzerland). The lymphocyte populations were analyzed after gating on the CD45^+^F4/80^- ^compartment. For intracellular staining, cells were incubated with antibodies against surface markers first, then fixed and permeabilized with Cytofix/Cytoperm kit from BD Biosciences (San Diego, CA). Cells were analyzed in a FACSCalibur (BD Biosciences, San Diego, CA). The number of acquired events is indicated in the figure legends. For cell cycle analyzes, TC-1 cells were harvested by tripsinization, fixed in 4% buffered formaldehyde and incubated in a buffer containing 0.1% (v/v) Triton X-100, 0.2 mg/ml RNase (DNase free) (Sigma, St Louis, MO) and 10 mg/ml propidium iodide (BD Biosciences, San Diego, CA) for 15 min at 37°C with gentle agitation. Cells were subsequently washed and analyzed in a FACSCalibur (BD Biosciences, San Diego, CA).

### Cell sorting

CD45^+ ^cells were sorted from total tumor suspensions by positive selection after incubation with biotin conjugated anti-CD45 and streptavidin conjugated magnetic beads (Miltenyi Biotec, Germany) and loading in columns exposed to magnetic field (MACS LS+ Separation Columns) (Miltenyi Biotec, Germany). The negative fraction was used as CD45^- ^population for RNA expression analyzes. In general, we obtained 95% pure cells with at least 90% viability.

Peritoneal macrophages were sorted from the peritoneal resident cells by positive selection with purified anti-F4/80 antibody, followed by anti-rat PE and and isolation using the EasySep anti-PE magnetic beads (Stem Cell Technologies, Vancouver, CA).

For protein expression experiments, tumors were digested as described before, and single cell suspensions were blocked with Fc blocking reagent and stained with FITC conjugated anti-CD11b, PE conjugated anti-Gr1, PECy5.5 conjugated anti-F4/80 and APC conjugated anti-CD45. Populations CD45^+^CD11b^+^F4/80^+ ^or CD45^+^CD11^+^Gr1^+ ^were sorted in a FACSCalibur (BD Biosciences, San Diego, CA). These sortings resulted in populations with 90-95% viability, approximately 90% purity for the F4/80^+ ^population, which is the most abundant in the tumors, and approximately 80% purity for the Gr1^+ ^population.

### Lymphocyte ex-vivo cultures

Total cell suspensions from peripheral lymph nodes were seeded in 24 well culture dishes, 1 × 10^6 ^cells/well in 10% fetal bovine serum and stimulated with 15 μg/ml each HPV16 E7_49-57 _peptide and HPV16 E6. After 5 days of incubation, cells were harvested, stained with anti-CD4 (cloneGK1.5), anti-CD8 (clone 53-6.7), fixed and stained with anti-Foxp3 (clone FJK-16S) using the FITC anti-rat/mouse Foxp3 staining set (eBioscience, San Diego, CA). Cells were analyzed by flow cytometry, when 100.000 events were acquired for posterior analyzes.

### RNA analysis

Cytokine expression analyses were performed by RT-PCR using RNA obtained from tumors collected 20 days post injection of TC-1 cells. Total RNA was extracted from infiltrating leukocytes (CD45^+^) and tumor cells (CD45^-^) using the RNeasy mini kit (Qiagen, Germany) according to the manufacture's recommended protocol. cDNA synthesis was performed using M-MLV reverse transcriptase (Invitrogen, Calrsbad, CA), oligo-dT and random primers, according to the manufacturer recommendation. We used 50 ng of cDNA in each amplification reaction. Expression of the constitutive gene HPRT was used as internal control. Amplified fragments were resolved in 1% agarose gels stained with 0.5 μg/ml ethidium bromide (Sigma-Aldrich, St Louis, MO).

Quantitative Real-time PCR analyses for IL-10 expression were done in CD45^+ ^and CD45^- ^cells isolated from TC-1 tumors. The reaction was performed in an ABI 7300 detection system (Applied Biosystems, Foster City, CA) with SYBR Green Mastermix (Applied Biosystems, UK) and specific primers for IL-10 (Forward CCCTGGGTGAGAAGCTGAAG and Reverse CACTGCCTTGCTCTTATTTTCACA) and GAPDH (Forward GGGCTGGCATTGCTCTCA and Reverse TGCTGTAGCCGTATTCATTGT). The relative quantification of IL-10 in comparison to a reference gene (GAPDH) was determined as described [35]. The relative expression ratio was calculated based on Real-time PCR efficiency and the crossing points for the IL-10 and GAPDH transcripts. The following formula was applied Ratio = E_IL-10_^(Ctcontrol-Ctsample)^/E_GAPDH_^(Ctcontrol-Ctsample)^, where E is the efficiency, Ctcontrol and Ctsample are the cycle numbers where GADPH and IL-10 amplification is detected above the threshold, respectively.

In order to analyze a panel of genes involved in the inflammatory response in our tumor model, Mouse Inflammatory Cytokines and Receptors RT^2 ^Profiler PCR Array System (PAMM-011A) were used (SABiosciences Corporation, Frederick, MD). RT^2 ^Profiler PCR Array System analysis was performed using cDNA obtained from total RNA of CD45^+ ^and CD45^-^sorted cells. Samples of cDNA were synthesized with M-MLV reverse transcriptase (Invitrogen, Carlsbad, CA), oligo-dT primer (IDT) and random primers. The reactions were performed on an ABI Prism 7300 sequence detection system (Applied Biosystems, Foster City, CA) using SYBR Green Mastermix (Applied Biosystems, UK). The mRNA expression levels were calculated as the ratio of the Ct averages of the housekeeping genes (HKG) and the Ct averages of the target gene (TG), where Ct (Cycle threshold) is the number of amplification cycles required for detection of fluorescence in a given cDNA sample. The amplification efficiencies of the targets (TG) and references (HKG) displayed no significant differences (data provided by SABiosciences Corporation, Fredrick, MD).

### Immunohistochemistry

Ice-cold acetone fixed 5 μm cryo-sections were blocked with 5% fetal bovine serum in PBS for 30 min at room temperature prior to incubation with biotinylated primary antibodies. The ABC Vectastain kit (Vector Laboraboratories, Burlingame, CA) was used for antibody detection, followed by Mayer's hematoxylin counterstaining and slide mounting in Permount (Fisher Scientific, Pittsburgh, PA). Images were obtained with an IX70 Olympus fluorescence microscope (Olympus, Corp. Tokyo, Japan), a DP70 Olympus camera, using its own software.

### Immunofluorescence

Acetone/methanol (2:1 v/v) fixed 5 μm cryo-sections were blocked with 5% fetal bovine serum in PBS for 30 min at room temperature prior to incubation with primary antibodies. Anti-CD8 antibody (clone 53-6.7, BD Biosciences, San Diego, CA), was incubated for 30 min after blocking. Tetramers (a kind donation from Dr. Luescher laboratory, Ludwig Institute for Cancer Research, Lausanne, Switzerland) were incubated for 2 hours at room temperature. Tissue was counterstained with 4'6-diamidino2-phenylindole (DAPI) (Sigma-Aldrich, Saint Louis, MO). For Granzyme B staining, sections were fixed in 4% formaldehyde after anti-CD8 staining and washing, washed, blocked and permeabilized with 5% FBS, 0.5% Triton X-100, 1 mg/ml rat IgG. Anti-Granzyme B (clone 16G6, eBiosciences, San Diego, CA) was incubated for 30 min at room temperature, followed by counterstaining with DAPI. Slides were mounted with Prolong (Invitrogen, Carlsbad, CA). Images were obtained with a BX61 Olympus fluorescence microscope (Olympus, Corp. Tokyo, Japan), a DP70 Olympus camera, using its own software.

### Cell cycle analysis

TC-1 cells were seeded in a density of 5 × 10^4 ^cells/well in 24 well plates in 10% calf serum supplemented RPMI. Cells were treated with 0 to 300 ng/ml of IL-10 for 4 days, after which cells were harvested by trypsinization. After washing in PBS, cells were fixed with 3.7% buffered formaldehyde for 24 hours at 4°C. Cells were washed twice with PBS and incubated for 45 min at 37°C in a 0.1% (v/v) Triton X-100, 200 μg/ml DNase free RNase A (Sigma, St Louis, MO), 10 μg/ml propidium iodide (BD Biosciences, San Diego, CA). Cells were washed once before acquisition in a FACSCalibur (BD Biosciences, San Diego, CA).

### Statistical analyses

Tumor growth kinetics experiments were tested by Mann-Whitney. Data from all other experiments were tested by t-test. In all cases, p < 0.05 was considered significant.

## Results

### Leukocytes infiltrating TC-1 tumors express IL-10

**Figure 1:**
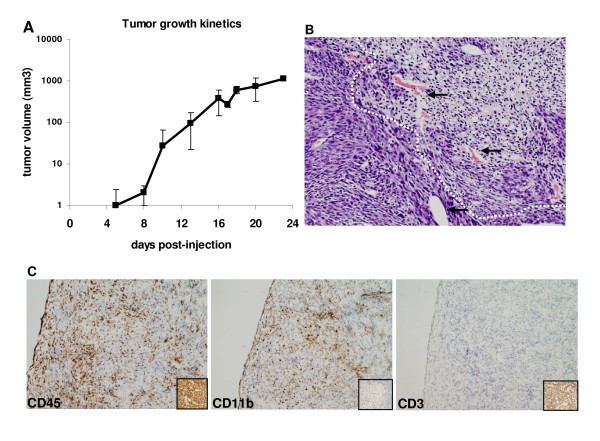
**Characterization of TC-1 derived tumors**. A. Tumor growth kinetics after injection of 10^5 ^TC-1 cells in the dorsal flank of C57BL/6 mice. Results are the average of tumor measures from 9 mice. B. Histology of TC-1 tumors. Tumor cryo-sections stained with hematoxylin/eosin exemplifying areas of leukocyte infiltrate, circulated by doted lines (100 × magnification). Arrows indicate some of the blood vessels. C. Immunohistochemistry characterization of TC-1 tumors. Tumor cryo-sections were incubated with antibodies against the indicated molecules. Antigen-antibody reaction was detected through peroxidase/DAB staining. Positive controls for each antibody are in the insets in each panel (200 × magnification).

TC-1 cells express E6 and E7 from HPV16 [34]. These two proteins are responsible for the maintenance of the transformed phenotype of HPV associated tumor cells, as well as for most of the immune evasion mechanisms displayed by these tumors [4]. TC-1 cells injected into C57BL/6 mice form tumors within 10 days after injection (Fig. 1A). Histological analysis of these tumors showed leukocyte infiltrate distributed all over the tumor area (Fig. 1B). This result was confirmed by immunohistochemistry showing CD45^+ ^cells representing the total leukocyte population, distributed throughout the tumor (Fig. 1C, left panel). We also observed that most of the infiltrate is of myeloid origin, CD11b^+ ^cells (Fig. 1C, middle panel). No T cells, identified by anti-CD3, were found in TC-1 tumors by this method (Fig. 1C, right panel). CD11b^+ ^tumor infiltrating cells may correspond to a number of populations from myeloid derived suppressor cells (MDSC) to tumor associated macrophages [27], or even granulocytes. Data from our laboratory indicate that most of these cells are tumor infiltrating macrophages with a M2-like phenotype [32].

**Figure 2:**
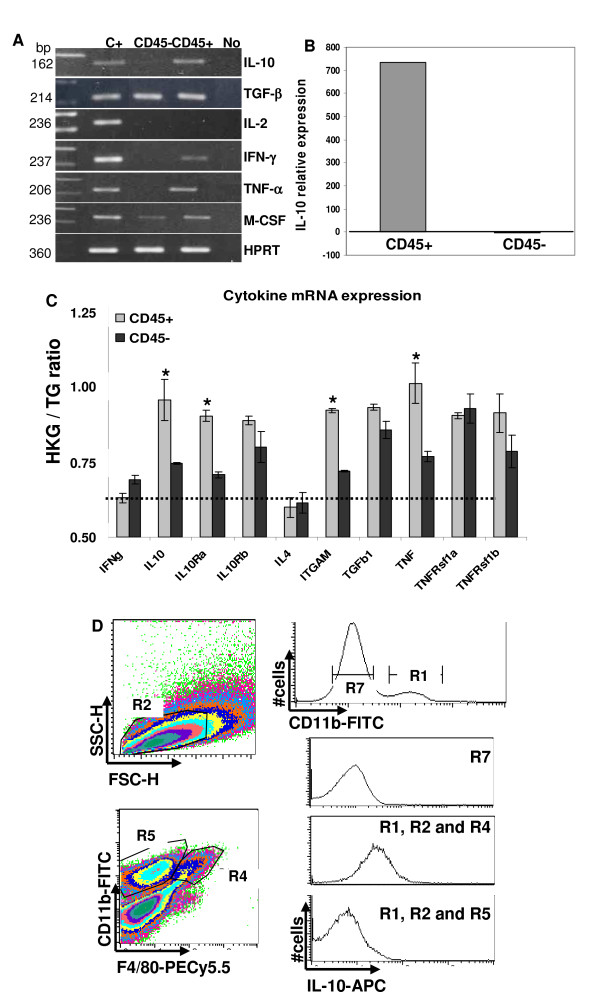
**Analysis of cytokine expression in TC-1 tumors**. RNA samples from CD45^+ ^and CD45^-^sorted cells were used for cytokine expression analysis. A. Qualitative cytokine mRNA expression analysis in total RNA samples from tumors 20 days post-injection. HPRT (Hypoxanthine Guanine Phosphoribosyltransferase) was used as control. **C+ **-positive control RNAs from cells stimulated with different cytokines. **No **-reactions without addition of cDNA. B. Quantitative Real-time PCR analysis of IL-10 expression in CD45^+ ^and CD45^- ^tumor fractions compared to RNA from total tumor population. Relative expression ratio is calculated based on ΔΔCt method. C. Results from SuperArray RT-PCR. Data is presented as the ratio of the housekeeping genes Ct (HKG) and the target gene Ct (TG). Ratios above 0.6 were defined as positive expression (dashed line). Light gray bars - CD45^+ ^cells cDNA, and dark gray bars - CD45^- ^cells cDNA. Asterisks indicate p < 0.05 tested by t-test comparing CD45^+ ^and CD45^- ^expression. D. IL-10 intracellular staining. Cell suspensions were stained with anti-CD11b and anti-F4/80 before fixation and with anti-IL-10 after fixation and permeabilization. FACS analysis was performed in a FACSCalibur, where the CD11b^+ ^population (top histogram, R1) was used to define the R2 region in the FSCxSSC plot. The CD11b × F4/80 plot was gated on R2 and the histograms representing IL-10 expression were gated on R1 and R2 and R4 or R5, CD11b^+^F4/80^+ ^macrophages or the CD11b^+ ^population, respectively; IL-10 expression in the tumor cells corresponds to cells gated on R7. Result representative of 3 independent experiments.

Tumor inflammatory infiltrate may contribute to tumor fate and immune responses against tumors [26-28]. Aiming to characterize the tumor environment and how the tumor infiltrating population influences the anti-tumor immune responses, we analyzed cytokine expression in the CD45^+ ^and CD45^- ^tumor cell populations. Initially, we investigated mRNA expression of cytokines by RT-PCR. We observed that TGFβ and M-CSF are expressed by both CD45^- ^tumor cells as by the CD45^+ ^infiltrate. Other cytokines were exclusively expressed by the leukocyte infiltrate: IL-10, TNFα and IFNγ (Fig. 2A). To quantify IL-10 expression, RNA samples from total tumor tissue, CD45^+ ^and CD45^- ^sorted cells were analyzed by real time PCR. Compared to the IL-10 expression in the total tumor population, CD45^+ ^cells produced 700 times more IL-10 mRNA than CD45^- ^cells (Fig. 2B). Accordingly, using the SuperArray RT-PCR platform, we observed significantly higher expression of IL-10 and its receptors chains correspondent mRNAs in CD45^+ ^than in CD45^- ^cell samples (Fig. 2C). Other genes that were more expressed in CD45^+ ^cells than in CD45^- ^cells were: Integrin αM (ITGAM), TNFα and TNF receptor b (TNFRsf1b). TGFβ and TNFRsf1a displayed high expression in both tumor cell compartments, while IL-4 and IFNγ had low expression levels (Fig. 2C).

Besides RNA expression, we also demonstrated IL-10 protein expression in tumor infiltrating macrophages. Fig. 2C shows IL-10 intracellular staining of TC-1 tumor cells (histogram gated on R7), CD11b^+ ^infiltrating population (histogram gated on R1 and R2 and R5) and on the macrophage population CD11^+^F4/80^+ ^(histogram gated on R1 and R2 and R4). The only population that expressed intracellular IL-10 was the macrophage population. We tested the CD11^+^Gr1^+ ^population, but this population was also negative for IL-10 expression (data not shown).

Our cytokine expression data indicate that TC-1 tumors infiltrating myeloid cells display a mixed cytokine profile including pro-inflammatory cytokines like TNFα, as well as potentially suppressive cytokines like IL-10 and TGFβ.

### IL-10 facilitates tumor growth

**Figure 3:**
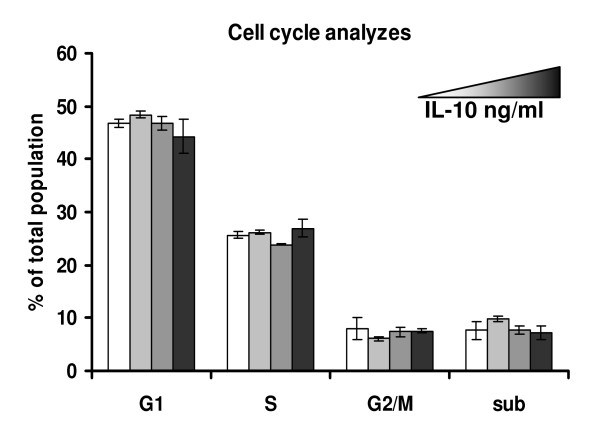
**Cell cycle analysis of TC-1 cells treated with IL-10**. A total of 25000 TC-1 cells were treated for 4 days with 0, 3, 30 or 300 ng/ml IL-10. After this period, cells were harvested, fixed, stained and analyzed in a FACSCalibur. A total of 50000 cells were acquired per sample (note that after 4 days in culture, TC-1 cells have exponentially grown, resulting in a total of about 1 million cells in the end of the experiment). No significant differences were observed between the different conditions.

**Figure 4:**
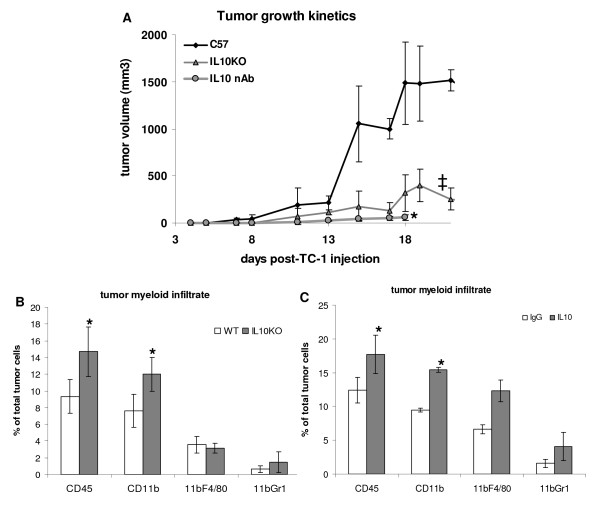
**Tumor growth and myeloid infiltrate in mice lacking IL-10 signaling**. A. TC-1 cells, 10^5 ^cells/mouse were injected into IL-10 KO (gray triangles), wild type C57BL/6 mice (black diamonds), C57BL/6 mice treated (intraperitoneal injections) with 10 μg/ml anti-IL-10 or 10 μg/ml anti-IL-10R in alternate doses, except for the first dose, which was anti-IL-10 together with anti-IL-10R in the same day as TC-1 cells were injected (gray circles) (some mice were treated in the same frequency with 500 μg of anti-IL-10R, clone 1b13a, included in the last group). The averages of tumor measurements of 12 mice, each condition, for the group of KO and wild type mice and 6 mice, each condition, for the group of antibody treated mice are represented; differences in tumor growth between control group C57BL/6 and IL-10 deficient mice (‡) or mice treated with neutralizing antibodies (*) were highly significant, in both cases p-value < 0.01. B and C. Analysis of tumor infiltrate by flow cytometry. Tumors were collected 15 or 17 days post injection. Total cell suspensions were stained with antibodies against the indicated markers and analyzed in a FACSCalibur. At least 30000 events were acquired per sample; * indicates significant differences between groups.

Macrophages associated to TC-1 tumors play a role in tumor growth. When we depleted this population from animals, using clodronate liposomes, we observed decreased tumor growth and an increase in expansion of CD8 specific T cells in the spleen, as well as infiltrating the tumors [32]. As we demonstrated, these macrophages express IL-10 (previous section and [32]). Due to the described roles of IL-10 in the literature, we raised the hypothesis that IL-10 may be involved in the mechanism by which this macrophage population facilitates tumor growth. We first tested whether IL-10 treatment had any effect on TC-1 proliferation *in vitro*. As shown in Fig. 3, increasing concentrations of recombinant IL-10 had no effect on TC-1 cell cycle, indicating that proliferation of TC-1 is not regulated by IL-10. We continued our investigation by injecting TC-1 cells in IL-10 deficient mice or mice treated with neutralizing antibodies against IL-10 and/or IL-10R (same effect was observed treating mice with alternate doses of 10 μg of anti-IL-10 and anti-IL-10R from R&D Systems or doses of 500 μg of the anti-IL-10R 1B13a monoclonal antibody purified in our laboratory). TC-1 tumor growth in IL-10 knockout was significantly slower than in wild type mice (Fig. 4A ‡). In mice treated with anti-IL-10 neutralizing antibodies, we also observed a significant reduction in tumor growth in comparison with C57BL/6 mice (Fig. 4A *). Significant reduction in tumor growth was also observed when anti-IL-10/IL-10R antibody treated mice were compared with tumor growth in mice treated with irrelevant IgG in the same amount, and doses, as the specific antibodies. This control group showed a partial effect in reducing tumor growth, which could be due to activation and recruitment of neutrophils (to be published elsewhere). Nevertheless, the mice treated with anti-IL10/IL10R antibodies had even slower tumor growth, with a significance of p = 0.0005.

Blocking of IL-10 signaling, via neutralization with antibody or due to gene knockout, led to a significant increase in CD11b^+ ^tumor infiltrating cells, which did not reflect a significant increase in numbers of TAM or CD11b^+^Gr1^+ ^cells (Fig. 4B and 4C).

IL-10 does not have any effect on TC-1 proliferation (Fig. 3). Therefore the inhibition of tumor growth should be due to the lack of the suppressive effect that IL-10 may have on anti-tumor immune responses.

### IL-10 decreases anti-tumor antigen T cell responses

IL-10 has the potential to suppress CD8 T cell responses and induce regulatory phenotype in T cells [36-38]. We next asked whether the decrease in tumor growth in IL-10 deficient mice or mice treated with neutralizing anti-IL-10/IL-10R had any effect on lymphocyte populations.

**Figure 5:**
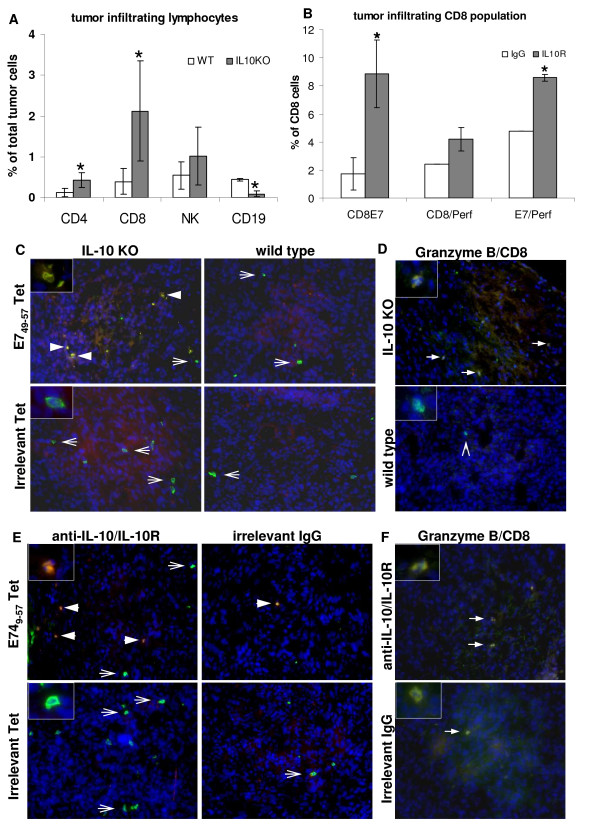
**Lymphocyte infiltrate in tumors from IL-10 deficient mice (A, C and D) or from anti-IL10/IL-10R neutralizing antibody treated mice (B, E and F)**. A. Single cell suspensions were stained with the indicated antibodies plus anti-CD45 and anti-F4/80. Lymphocytes were analyzed in the CD45^+^F4/80^- ^population. B. After 6 hours incubation in tissue culture flasks, non adherent cells from total tumor suspension were harvested and stained with the indicated antibodies, tetramer and anti-CD45. C and E. Immunofluorescence of cryo-sections from tumors harvested from IL-10 KO and wild type mice or neutralizing anti-IL10/IL-1R and irrelevant IgG antibodies, as indicated. Sections were stained with anti-CD8 (in FITC, green) and PE (red) conjugated HPV16 E7_49-57 _tetramer - E7_49-57 _Tet, and irrelevant tetramer - Irrelevant Tet. Cells were counterstained with DAPI (blue). Arrowheads indicate double positive cells in yellow, arrows indicate cells positive only for CD8. D and F. Granzyme B expression in tumor cryo-sections from IL-10 KO and wild type mice or mice treated with anti-IL10/IL-10R neutralizing antibodies and irrelevant IgG, as indicated. Sections were stained with anti-CD8 (green), followed by fixation, permeabilization and staining with anti-Granzyme B (red). Tissue was counterstained with DAPI. Solid arrows indicate double positive cells, arrowhead indicate CD8 only positive cell. Insets show details of cells positive only for CD8 (green) or double positive CD8/tetramer (C and E), CD8/Granzyme B (D and F) in yellow or orange due to the sobreposition of emission from FITC and PE (tetramer) or alexa 594 (Granzyme B).

First, we observed a significant increase in CD4 and CD8 cell numbers in tumors from IL10 deficient mice and mice treated with anti-IL-10/IL-10R neutralizing antibodies. In IL-10 deficient mice, there was 3.6 fold more CD4 lymphocytes and 4.2 fold more CD8 lymphocytes than what was found in tumors growing in wild type mice, and a decrease of 3.5 fold in the B cell population in the same tumors (Fig. 5A, asterisks indicate significant differences between wild type and IL-10 deficient mice). In mice treated with anti-IL10/IL-10R neutralizing antibodies compared to controls, we observed a 5 fold increase in the CD8 infiltrating cells specific for the E7_49-57 _peptide (Fig. 5B), an epitope previously described as the immunodominant epitope presented by TC-1 cells in the MHC-I context. Approximately 2 fold increase in the number of double positive CD8 Perforin^+ ^E7^+ ^T cells was observed in the IL-10R neutralized mice compared to controls (Fig. 5B), indicating that absence of IL-10 signaling enhances tumor infiltration by potentially cytotoxic T cells. These results were confirmed to some extent by staining tumor cryo-sections with tetramers and anti-CD8 and anti-Granzyme B antibodies. Figs. 5C and 5E show CD8 and tetramer staining for IL10 deficient mice and mice treated with anti-IL10/IL-10R, respectively. And Figs. 5D and 5F, show the same tumors stained with anti-CD8 and anti-Grazyme B. As observed previously, whenever IL-10 activity was blocked, we observed more CD8 E7 specific T cells infiltrating the tumors (Fig. 5C and 5E, arrowheads), while some nonspecific T cells were also detected (Fig. 5C and 5E, arrows). We did not observe binding of tetramer loaded with an irrelevant peptide to any of the tumors. Granzyme B expression, another marker of cytotoxic activity, was detected in larger number in tumors from mice with the IL-10 signaling blocked (Fig. 5D and 5F, arrows).

These results indicate that lymphocytes migrate to TC-1 tumors when IL-10 signaling is blocked. Whether the migration is due to altered chemokine expression, more efficiently activated antigen presenting cells or simply activation of T cell responses, or more likely a combination of these effects, is still to be determined. As regulatory T cells play a role in human cervical tumors [20] and as we had previous indication that tumor associated macrophages induced regulatory phenotype on T cells [32], we investigated if IL-10 may have a role in expansion of regulatory T cells in our model.

**Figure 6:**
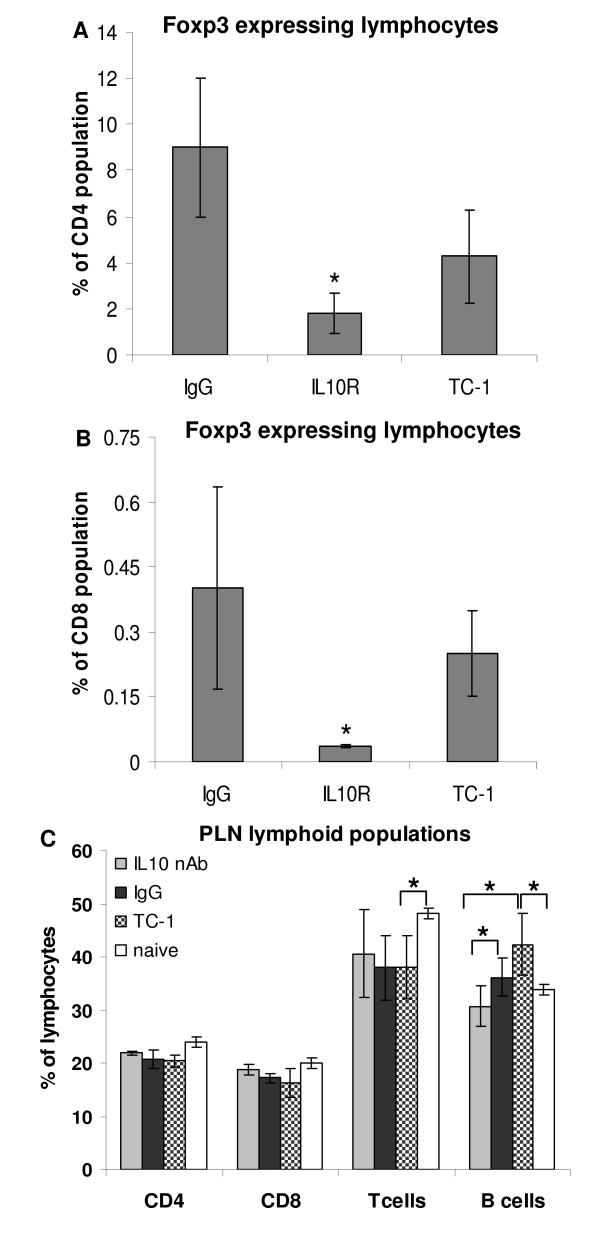
**Induction of regulatory phenotype on T cells depends on IL-10**. A and B. Lymph node cultures were stimulated with HPV16 E6 and E7 peptides, 15 μg each, for 6 days. Cells were harvested and stained for CD4 (A), CD8 (B) and Foxp3 and analyzed by flow cytometry. For each sample, 10^5 ^lymphocytes were acquired. Lymph nodes were harvested from irrelevant IgG treated mice (IgG), anti-IL10R treated mice (IL10R) or TC-1 tumors bearing mice untreated (TC-1) 18 days after TC-1 cells injection. C. Flow cytometry analysis of peripheral lymph node suspensions stained with the indicated antibodies. Lymph node single cell suspensions were stained with antibodies against the indicated markers and analyzed by flow cytometry. At least 30000 events were acquired per condition. The results are the average of 6 mice. Naïve are cells obtained from mice never exposed to TC-1 tumors; TC-1 corresponds to cells obtained from untreated mice injected with TC-1 cells; IgG and IL-10 correspond to cells obtained from mice with tumors treated with irrelevant IgG and anti-IL-10 and anti-IL-10R, respectively. Asterisks indicate p < 0.05 in t-test.

Using E7 and E6 peptides as antigens, we observed that stimulated lymphocytes from mice treated with irrelevant IgG or wild type mice with TC-1 tumors had a higher proportion of Foxp3^+ ^CD4^+ ^and Foxp3^+^CD8^+ ^cells than mice treated with anti-IL-10R antibody (Fig. 6A and 6B). Foxp3 is a marker of regulatory lymphocytes, which function is to inactivate effector T cells. Our results indicate that, at least in part, IL-10 facilitates tumor growth by inducing T cell regulatory phenotype and therefore inhibiting T cell anti-tumor activity.

As mentioned before, less B cells infiltrate tumors of IL-10 deficient mice. We observed that draining lymph nodes of mice with TC-1 tumors have a lower T/B cell ratio than naïve mice. When IL-10 is neutralized, the T/B cell ratio in the lymph nodes is similar to the naïve mice observed ratios (Fig. 6C), an indication that IL-10 may have a role in proliferation and homing of B cells in mice bearing TC-1 tumors.

## Discussion

Several factors influence tumor fate, among them the immune responses against tumor antigens. In the case of tumors caused by viruses, viral antigens may be recognized by the host. However, viruses as well as tumor cells display several mechanisms of immune evasion. Among them, the expression of suppressor cytokines like IL-10 has been described in many malignancies [15,16], including tumors and tumor precursor lesions associated to HPV [21-23]. Our laboratory has been working on the characterization of the leukocyte population infiltrating the mouse tumor model TC-1. Previous work from our laboratory show that most of the TC-1 tumor infiltrating leukocytes are M2-like macrophages and that depletion of this population inhibits tumor growth and enhances antitumor responses, showing a clear role of this population in facilitating tumor growth [32]. Moreover, these macrophages induce IL-10 and Foxp3 expression on lymphocytes [32]. Accordingly, depletion of the tumor associated macrophage population led to an increase in tumor infiltration by HPV16 specific CD8 T cells and expansion of this population in the spleen [32].

In this work, we first showed that the CD45^+ ^tumor fraction expressed different cytokines, among them IL-10, TNFα and TGFβ. Although several cytokines are expressed by tumor macrophages and may have a potential role in tumor growth, this work focuses on IL-10, due to its role as a regulator of T cell activity. TC-1 tumor cells expressed TGFβ, but not IL-10. They did not proliferate in response to IL-10 either (Fig. 3), indicating that in our model, IL-10 does not act as a mitogen to tumor cells. However, a correlation between IL-10 increased expression and HPV associated lesions progression to high grade lesions have been well described in the literature [9-11,21-23]. Moreover, women with cervical cancer have circulating regulatory T cells that inhibit effector response and secrete IL-10 upon antigen stimulation [20]. The molecular mechanisms underlying this correlation have not been characterized until now. Due to the described roles of IL-10 in the literature, like suppression of CD8 function [37-40] and increase in B cell proliferation [41,42], as well as evidence from our laboratory showing the role of tumor associated macrophages in tumor growth, we decided to investigate if this cytokine is part of the immune evasion mechanism displayed by E6/E7 transformed cells.

In this study, we observed a decrease of tumor growth in mouse models where IL-10 signaling was blocked: IL-10 deficient mice or mice treated with anti-IL-10/anti-IL-10R neutralizing antibodies. Prolonged survival and a decrease in tumor development were observed in other in vivo models using IL-10 deficient animals [43]. Moreover, transgenic mice expressing IL-10 show accelerated tumor growth that can be diminished by IL-10 neutralizing antibody [44,45]. We realize that the TC-1 cell line is transformed with high risk HPV16 oncogenes E6/E7 and EJ-ras [34]. Ras-associated tumors may induce IL-10 expression as a form of evasion. Indeed, this seems to be the case of some colon cancers [17]. On the other hand, thyroid tumors, which frequently display mutation in ras, respond to an autocrine IL-10 signalization loop where tumor cells secrete and proliferate in response to IL-10 [19]. In colon cancer, although there is discussion in the literature, IL-10 may be used as therapy once it could counter-act the action of pro-inflammatory cytokines that cause chronic inflammation leading to cancer [46]. In our model, TC-1 cells do not secrete or proliferate in response to IL-10. Therefore the role IL-10 plays on TC-1 tumor growth is probably associated only to the regulation of immune responses and its source, as demonstrated in this work, is the myeloid tumor infiltrate.

In parallel to a reduction in tumor growth, we observed in IL-10 deficient mice or mice treated with anti-IL-10/anti-IL-10R neutralizing antibodies that there was an increase tumor infiltration by CD4 and CD8 lymphocytes, which were rare in tumors from control wild type mice. Importantly, there was a significant increase in CD8 E7 specific cells with a cytotoxic phenotype in mice that had the IL-10 signaling blocked compared to controls. When stimulated with E6 and E7 peptides, lymphocytes from control mice and mice treated with irrelevant IgG had a significantly higher proportion of Foxp3 regulatory cells than lymphocytes from mice with neutralized IL-10 signaling. This result indicates that IL-10 suppresses T cell activity by inducing a regulatory phenotype on CD4 and CD8 lymphocytes. We cannot, however, rule out a possible effect of tumor microenvironment that would facilitate T cell recruitment. Probably, a combination of both events occurs in our model. There was, for example, a significant increase in the CD11b^+ ^myeloid population in IL-10 deficient mice and mice treated with anti-IL-10/IL-10R neutralizing antibodies when compared to controls, indicating a difference in the tumor microenvironment due to IL-10 depletion. In HPV16 tumor models, a role for CCL2 in the recruitment of macrophages for the tumor site was described by Pahler and collaborators [47]. In our laboratory, using a keratinocyte cell line expressing HPV16 E6/E7 and its parental cell line, we were able to show that the CCL2 effect on myeloid cell recruitment is dependent on E6/E7 expression [Bolpetti in preparation]. Interestingly, IL-10 inhibits CCL2 expression 48, in a way that it is possible that the increase in CD11b^+ ^cells that infiltrate tumors from either IL-10 deficient mice or mice treated with neutralizing anti-IL10R antibody may be due to an increase in CCL2 expression.

Another effect of IL-10 neutralization in mice was the decrease in tumor cell infiltration by B cells and reduction of the B cell numbers in peripheral lymph nodes. This result is not surprising since one described role for IL-10 is B cell proliferation [41,42]. It is possible that IL-10 causes a skewing in adaptive response toward humoral response, which may contribute to the inhibition of T cell function or simply which is ineffective against tumor cells.

## Conclusions

IL-10 production by tumor associated myeloid cells, mainly macrophages, induces expansion of HPV specific regulatory T cells, possibly inhibiting effector T cell anti-tumor activity. We believe our work suggests that this is one of the possible mechanisms behind the epidemiologic data correlating HPV associated tumor progression and increase in IL-10 expression.

## Abbreviations

E6: HPV early protein/gene 6; E7: HPV early protein/gene 7; FBS: fetal bovine serum; HPV: Human Papillomavirus; HPV16: Human Papillomavirus type 16; IL-10: Interleukin-10; PBS: phosphate buffered saline; TGFβ: Tumor Growth Factor β; TNFα: Tumor Necrosis Factor α.

## Authors' contributions

All authors have read and agreed to the contents of this manuscript. AB conducted the tumor characterization, RNA analyzes and knockout mice studies. JSS donated the IL-10 deficient mice and provided infra-structure for the knockout experiments, as well as a critical review of the manuscript. LLV is the head of the Virology laboratory at the São Paulo branch of the Ludwig Institute for Cancer Research, where most of this work was developed and contributed with the experimental design, as well as critical review of this manuscript. APL is the mentor of this project, did the neutralizing antibody experiments, T cell activity and phenotype characterization and IL-10 intracellular staining experiments. APL also wrote this manuscript.

## References

[B1] IARC-International Agency for Research on Cancer-World Health Organization IARC Monographs on the evaluation of carcinogenic risks to humans: human papillomaviruses 2007 90 Lyon: Human papillomavirus PMC4781057 18354839

[B2] Schlecht NF Kulaga S Robitaille J Ferreira S Santos M Miyamura RA Duarte-Franco E Rohan TE Ferenczy A Villa LL Franco EL Persistent human papillomavirus infection as a prediction of cervical intraepithelial neoplasia JAMA 2001 286 3106 3114 10.1001/jama.286.24.3106 11754676

[B3] Schlecht NF Platt RW Duarte-Franco E Costa MC Sobrinho JP Prado JC Ferenczy A Rohan TE Villa LL Franco EL Human papillomavirus infection and time to progression and regression of cervical intraepithelial neoplasia J Natl Cancer Inst 2003 95 1336 1343 12953088 10.1093/jnci/djg037

[B4] Tindle RW Immune evasion in human papillomavirus-associated cervical cancer Nat Rev Cancer 2002 2 59 65 10.1038/nrc700 11902586

[B5] Um SJ Rhyu JW Kim EJ Jeon KC Hwang ES Park JS Abrogation of IRF-1 response by high-risk HPV E7 protein in vivo Cancer Lett 2002 179 205 212 11888675 10.1016/s0304-3835(01)00871-0

[B6] Park JS Kim EJ Kwon HJ Hwang ES Namkoong SE Um SJ Inactivation of interferon regulatory factor-1 tumor suppressor protein by HPV E7 oncoprotein. Implication for the E7-mediated immune evasion mechanism in cervical carcinogenesis J Biol Chem 2000 275 6764 6769 10.1074/jbc.275.10.6764 10702232

[B7] Xu Q Wang S Xi L Wu S Chen G Zhao Y Wu Y Ma D Effects of human papillomavirus type 16 E7 protein on the growth of cervical carcinoma cells and immuno-escape through the TGF-beta 1 signaling pathway Gynecol. Oncol 2000 101 132 139 10.1016/j.ygyno.2005.09.051 16269171

[B8] Hazelbag S Kenter GG Gorter A Fleuren GJ Prognostic relevance of TGF-beta 1 and PAI-1 in cervical cancer Int J Cancer 2004 112 1020 1028 10.1002/ijc.20512 15386352

[B9] Chopra V Dinh TV Hannigan EV Circulating serum level of cytokines and angiogenic factors in patients with cervical cancer Cancer Invest 1997 16 152 159 10.3109/07357909809050029 9541628

[B10] Jacobs N Giannini SL Doyen J Baptista A Moutschen M Boniver J Delvenne P Inverse modulation of IL-10 and IL-12 in the blood of women with preneoplastic lesions of the uterine cervix Clin Exp Immunol 1998 111 219 224 10.1046/j.1365-2249.1998.00437.x 9472685 PMC1904851

[B11] Clerici M Merola M Ferrario E Trabattoni D Villa ML Stefanon B Venzon DJ Shearer GM De Palo G Clerici E Cytokine production patterns in cervical intraepithelial neoplasia: association with human papillomavirus infection J Natl Cancer Inst 1997 89 245 250 10.1093/jnci/89.3.245 9017005

[B12] Salazar-Onfray F López MN Mendoza-Naranjo A Paradoxical effects of cytokines in tumor immune surveillance and tumor immune escape Cytokine Growth Factor Rev 2007 18 171 182 10.1016/j.cytogfr.2007.01.015 17329145

[B13] Matsuda M Salazar F Petersson M Masucci G Hansson J Pisa P Zhang QJ Masucci MG Kiessling R Interleukin 10 pretreatment protects target cells from tumor-and allo-specific cytotoxic T cells and downregulates HLA class I expression J Exp Med 1994 180 2371 2376 10.1084/jem.180.6.2371 7964510 PMC2191780

[B14] Petersson M Charo J Salazar-Onfray F Noffz G Mohaupt M Qin Z Klein G Blankenstein T Kiessling R Constitutive IL-10 production accounts for the high NK sensitivity, low MHC class I expression, and poor transporter associated with antigen processing (TAP)-1/2 function in the prototype NK target YAC-1 J Immunol 1998 161 2099 2105 9725200

[B15] Sato T McCue P Masuoka K Salwen S Lattime EC Mastrangelo MJ Berd D Interleukin 10 production by human melanoma Clin Cancer Res 1996 2 1383 1390 9816311

[B16] Fortis C Foppoli M Gianotti L Galli L Citterio G Consogno G Gentilini O Braga M Increased interleukin-10 serum levels in patients with solid tumours Cancer Lett 1996 104 1 5 10.1016/0304-3835(96)04213-9 8640735

[B17] Herbeuval J Lelievre E Lambert C Dy M Genin C Recruitment of STAT3 for Production of IL-10 by Colon Carcinoma Cells Induced by Macrophage-Derived IL-6 J Immunol 2004 172 4630 36 15034082 10.4049/jimmunol.172.7.4630

[B18] Bellone G Turletti A Artusio E Mareschi K Carbone A Tibaudi D Robecchi A Emanuelli G Rodeck U Tumor-Associated Transforming Growth Factor-band Interleukin-10 Contribute to a SystemicTh2 Immune Phenotype in Pancreatic Carcinoma Patients Am J Pathol 1999 155 537 547 10433946 10.1016/s0002-9440(10)65149-8 PMC1866873

[B19] Todaro M Zerilli M Ricci-Vitiani L Bini M Alea MP Florena AM Miceli L Condorelli C Bonventre S Di Gesu' G De Maria R Stassi G Autocrine Production of Interleukin-4 and Interleukin-10 Is Required for Survival and Growth of Thyroid Cancer Cells Cancer Res 2006 66 1491 9 10.1158/0008-5472.CAN-05-2514 16452205

[B20] de Jong A van Poelgeest MI van der Hulst JM Drijfhout JW Fleuren GJ Melief CJ Kenter G Offringa R van der Burg SH Human papillomavirus type-16-positive cancer is associated with impaired CD4+ T cell immunity against early antigens E2 and E6 Cancer Res 2004 64 5449 5455 10.1158/0008-5472.CAN-04-0831 15289354

[B21] Bermudez-Morales VH Gutierrez LX Alcocer-Gonzalez JM Burguete A Madrid-Marina V Correlation between IL-10 gene expression and HPV infection in cervical cancer: a mechanism for immune response escape Cancer Invest 2008 26 1037 1043 10.1080/07357900802112693 18798072

[B22] Mindiola R Caulejas D Nùñez-Troconis J Araújo M Delgado M Mosquera J Increased number of IL-2, IL-2 receptor and IL-10 positive cells in premalignant lesions of the cervix Invest Clin 2008 49 533 545 19245171

[B23] Azar KK Tani M Yasuda H Sakai A Inoue M Sasagawa T Increased secretion patterns of interleukin-10 and tumor necrosis factor-alpha in cervical squamous intraepithelial lesions Hum Pathol 2004 35 1376 1384 10.1016/j.humpath.2004.08.012 15668895

[B24] Zoodsma M Nolte IM Schipper M Oosterom E van der Steege G de Vries EG Te Meerman GJ van der Zee AG Interleukin-10 and Fas polymorphisms and susceptibility for (pre) neoplastic cervical disease Int J Gynecol Cancer 2005 3 282 290 10.1111/j.1525-1438.2005.00433.x 16343245

[B25] Shrestha S Wang C Aissani B Wilson CM Tang J Kaslow RA Interleukin-10 gene (IL10) polymorphisms and human papillomavirus clearance among immunosuppressed adolescents Cancer Epidemiol Biomarkers Prev 2007 16 1626 1632 10.1158/1055-9965.EPI-06-0881 17684137

[B26] Mantovani A Sozzani S Locati M Allavena P Sica A Macrophage polarization: tumor-associated macrophages as a paradigm for polarized M2 mononuclear phagocytes TRENDS Immunol 2002 23 549 555 10.1016/S1471-4906(02)02302-5 12401408

[B27] Sica A Bronte V Altered macrophage differentiation and immune dysfunction in tumor development J Clin Invest 2007 117 1155 1166 10.1172/JCI31422 17476345 PMC1857267

[B28] Sica A Saccani A Bottazzi B Polentarutti N Vecchi A van Damme J Mantovani A Autocrine production of IL-10 mediates defective IL-12 production and NF-kappa B activation in tumor-associated macrophages J Immunol 2000 15 762 767 10.4049/jimmunol.164.2.762 10623821

[B29] Hammes LS Tekmal RR Naud P Edelweiss MI Kirma N Valente PT Syrjänen KJ Cunha-Filho JS Macrophages, inflammation and risk of cervical intraepithelial neoplasia (CIN) progression-clinicopathological correlation Gynecol Oncol 2007 105 57 65 10.1016/j.ygyno.2006.11.023 17229459

[B30] Mazibrada J Rittà M Mondini M De Andrea M Azzimonti B Borgogna C Ciotti M Orlando A Surico N Chiusa L Landolfo S Gariglio M Interaction between inflammation and angiogenesis during different stages of cervical carcinogenesis Gynecol Oncol 2007 108 112 120 10.1016/j.ygyno.2007.08.095 17936343

[B31] Kobayashi A Weinberg V Darragh T Smith-McCunne K Evolving immunosuppressive microenvironment during human cervical carcinogenesis Mucosal Immunol 2008 1 412 20 10.1038/mi.2008.33 19079205

[B32] Lepique AP Daghastanli KR Cuccovia IM Villa LL HPV16 tumor associated macrophages suppress antitumor T cell responses Clin Cancer Res 2009 15 4391 400 10.1158/1078-0432.CCR-09-0489 19549768

[B33] Kuhn R Löhler J Rennick D Rajewsky K Müller W Interleukin-10-deficient mice develop chronic enterocolitis Cell 1993 75 263 274 10.1016/0092-8674(93)80068-P 8402911

[B34] Lin KY Guarnieri FG Staveley-O'Carroll KF Levitsky HI August JT Pardoll DM Wu TC Treatment of established tumors with a novel vaccine that enhances major histocompatibility class II presentation of tumor antigen Cancer Res 1996 56 21 26 8548765

[B35] Pfaffl MW A new mathematical model for relative quantification in real-time RT-PCR Nucleic Acids Res 2001 29 e45 10.1093/nar/29.9.e45 11328886 PMC55695

[B36] Biswas SK Sica A Lewis CE Plasticity of macrophage function during tumor progression regulation by distinct molecular mechanisms J Immunol 2008 180 2011 2017 18250403 10.4049/jimmunol.180.4.2011

[B37] Biswas PS Pedicord V Ploss A Menet E Leiner I Pamer EG Pathogen-specific CD8 T cell responses are directly inhibited by IL-10 J Immunol 2007 179 4520 4528 17878348 10.4049/jimmunol.179.7.4520

[B38] Loser K Apelt J Voskort M Mohaupt M Balkow S Schwarz T Grabbe S Beissert S IL-10 controls ultraviolet-induced carcinogenesis in mice J Immunol 2007 179 365 371 17579057 10.4049/jimmunol.179.1.365

[B39] Denning TL Wang YC Patel SR Williams IR Pulendran B Lamina propria macrophages and dendritic cells differentially induce regulatory and interleukin- 17 producing T cell responses Nat Immunol 2007 8 1086 94 10.1038/ni1511 17873879

[B40] Halak BK Maguire HC Lattime EC Jr Tumor-induced interleukin-10 inhibits type 1 immune responses directed at a tumor antigen as well as a non-tumor antigen present at the tumor site Cancer Res 1999 15 911 917 10029084

[B41] Itoh K Hirohata S The role of IL-10 in human B cell activation, proliferation and differentiation J Immunol 1995 154 4341 4350 7722292

[B42] Banchereau J Brière F Liu YJ Rousset F Molecular control of B lymphocyte growth and differentiation Stem Cells 1994 12 278 288 10.1002/stem.5530120304 7521239

[B43] Rankin EB Yu D Jiang J Shen H Pearce EJ Goldschmidt MH Levy DE Golovkina TV Hunter CA Thomas-Tikhonenko A An essential role of Th1 responses and interferon gamma in infection-mediated suppression of neoplastic growth Cancer Biol Ther 2003 2 687 693 14688478

[B44] Hagenbaugh A Sharma S Dubinett SM Wei SH Aranda R Cheroutre H Fowell DJ Binder S Tsao B Locksley RM Moore KW Kronenberg M Altered immune responses in interleukin 10 transgenic mice J Exp Med 1997 16 2101 2110 10.1084/jem.185.12.2101 PMC2196349 9182682

[B45] Kokura S Yoshida N Ishikawa T Higashihara H Sakamoto N Takagi T Uchiyama K Naito Y Mazda O Okanoue T Yoshikawa T Interleukin-10 plasmid DNA inhibits subcutaneous tumor growth of Colon 26 adenocarcinoma in mice Cancer Lett 2005 218 171 9 10.1016/j.canlet.2004.07.026 15670894

[B46] Pahler JC Tazzyman S Erez N Chen YY Murdoch C Nozawa H Lewis CE Hanahan D Plasticity in tumor-promoting inflammation: impairment of macrophage recruitment evokes a compensatory neutrophil response Neoplasia 2008 10 329 40 18392134 10.1593/neo.07871 PMC2288539

[B47] Stoffels B Schmidt J Nakao A Nazir A Chanthaphavong RS Bauer AJ Role of interleukin 10 in murine postoperative ileus Gut 2009 58 648 60 10.1136/gut.2008.153288 19359433

